# Viral Determinants and Vector Competence of Zika Virus Transmission

**DOI:** 10.3389/fmicb.2018.01040

**Published:** 2018-05-23

**Authors:** Hong-Wai Tham, Vinod Balasubramaniam, Man K. Ooi, Miaw-Fang Chew

**Affiliations:** ^1^Biology Research Laboratory, Faculty of Pharmacy, SEGi University, Petaling Jaya, Malaysia; ^2^Jeffrey Cheah School of Medicine and Health Sciences, Monash University Malaysia, Subang Jaya, Malaysia; ^3^Centre for Virus and Vaccine Research, School of Science and Technology, Sunway University, Subang Jaya, Malaysia

**Keywords:** Zika virus, arboviruses, mosquitoes, viral determinants, vector competence

## Abstract

Zika virus (ZIKV) has emerged as a new global health threat. Since its first discovery in Zika forest in Uganda, this virus has been isolated from several mosquito species, including *Aedes aegypti* and *Aedes albopictus*. The geographical distribution of these mosquito species across tropical and subtropical regions has led to several outbreaks, including the recent pandemic in Brazil, followed by the Pacific islands and other areas of North and South America. This has gained attention of the scientific community to elucidate the epidemiology and transmission of ZIKV. Despite its strong attention on clinical aspects for healthcare professionals, the relationships between ZIKV and its principal vectors, *A. aegypti* and *A. albopictus*, have not gained substantial interest in the scientific research community. As such, this review aims to summarize the current knowledge on ZIKV tropism and some important mechanisms which may be employed by the virus for effective strategies on viral survival in mosquitoes. In addition, this review identifies the areas of research that should be placed attention to, for which to be exploited for novel mosquito control strategies.

## Zika Virus

**FIGURE 1:**
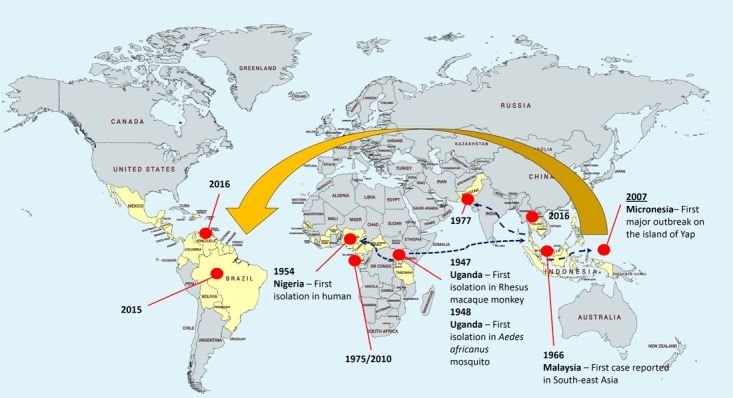
Zika virus epidemiology. The yellow area depicts the geographical distribution or landmarks of Zika virus. The virus was first isolated in Uganda from rhesus macaque monkey and *Aedes africanus* mosquito, followed by first isolation in human in 1954 in Nigeria. The subsequent circulations were restricted to Africa and Southeast Asia. In 2015, emergence of Zika virus in South America issued an alert to Pan American Health Organization (PAHO).

Zika virus (ZIKV) was first isolated from a rhesus macaque monkey in the Zika Forest of Uganda in 1947 (Dick et al., 1952), followed by the first virus isolation from *Aedes africanus* mosquito in the year after (Lanciotti et al., 2008). The first human infection was reported in Nigeria in 1954 (MacNamara, 1954), until the recent outbreak in Brazil in May 2015 (Rodriguez-Morales, 2015; Zanluca et al., 2015), followed by 29 other countries reported ZIKV transmission before 2016 (Hennessey, 2016; **Figure 1**). Soon after, the potential association of microcephaly to the neonates of ZIKV-infected mothers was reported (de Araújo et al., 2016; Mlakar et al., 2016). This was further supported by subsequent studies where infants with microcephaly were associated with ZIKV infection during pregnancy (de Paula Freitas et al., 2016; Ventura et al., 2016). However, to date, the specific associations between ZIKV and microcephaly remain plausible and no consensus was made.

**FIGURE 2:**
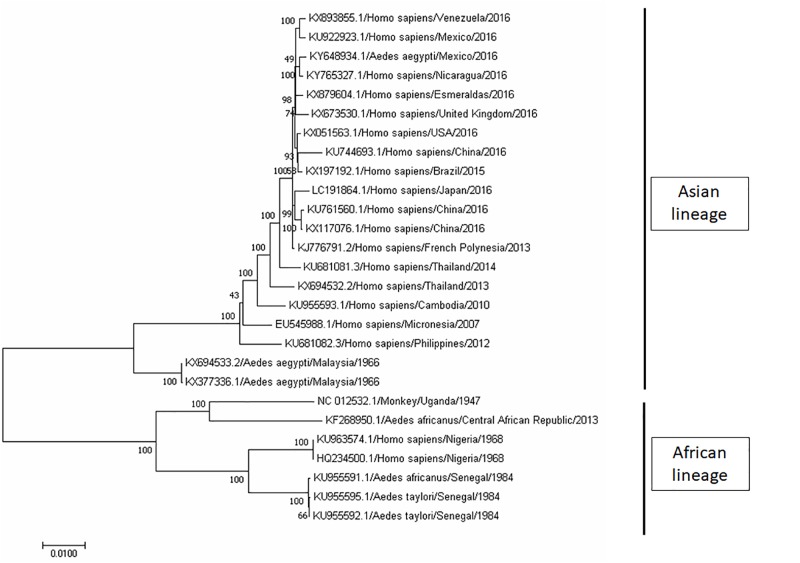
Phylogenetic tree of Zika virus (ZIKV). The complete genome sequences of ZIKV isolates were obtained from GenBank. The phylogenetic tree was constructed by maximum likelihood model with 1000 bootstrap replicates. The identification of the strains is in the format of accession number/host/country of isolation/year of isolation. ZIKV strains were grouped into two lineage, namely, Asian lineage and African lineage based on their geographical distribution.

ZIKV is an icosahedral, enveloped, single-stranded RNA virus (Heinz and Stiasny, 2017; Shi and Gao, 2017). It belongs to the *Flavivirus* genus, and the envelope consists of lipid bilayer and envelope glycoproteins (Heinz and Stiasny, 2017; Shi and Gao, 2017). Phylogenetic analyses clearly indicated that ZIKV can be grouped into two distinct lineages – Asian lineage and African lineage, based on their complete genome sequences obtained from National Center for Biotechnology Information (NCBI) and analyses using Molecular Evolutionary Genetic Analysis (MEGA) software (**Figure 2**). In addition, the evolutionarylineages changing over time as shown in **Figure 2** support the geographical distribution of ZIKV from 1947 (Uganda) to its first reported isolation as non-African lineage in 1969 (Malaysia; Marchette et al., 1969), until the recent widespread epidemic of Zika fever in 2015 (from Brazil to North and South America; **Figure 2**). Despite its low prevalence before 2015, the geographic distribution of ZIKV has been intensively studied through seroprevalence surveys. In Uganda, although ZIKV was found in *A. africanus*, a local mosquito strain, antibody prevalence in the residents of the same area was low (Dick, 1952; Dick et al., 1952). In the next 20 years, a large number of serological studies were recorded on the dynamic distribution of ZIKV from Africa (Smithburn, 1952; MacNamara, 1954; Smithburn et al., 1954; Robin and Mouchet, 1975; Jan et al., 1978; Saluzzo et al., 1982; Adekolu-John and Fagbami, 1983; Monlun et al., 1993) to Asia (Smithburn et al., 1954; Hammon et al., 1958; Pond, 1963; Darwish et al., 1983; Heang et al., 2012).

Similar to other important human-pathogenic arboviruses, such as yellow fever virus (YFV), dengue virus (DENV), and Japanese encephalitis virus (JEV), ZIKV maintains human-to-human transmission cycles through *Aedes* mosquitoes as the vector (Weaver and Reisen, 2010). In addition to vector transmission, the potential for sexual ZIKV transmission was first reported in 2015 whereby the virus was isolated from a male patient (Musso et al., 2015). Soon after, a health report released in Texas confirmed such transmission mode of ZIKV (McCarthy, 2016), which currently appears to be the only known arbovirus linked to this transmission mode in humans. This report was supported by Govero et al. (2016), whereby *in vivo* ZIKV infection was observed in the spermatogonia, spermatocytes, and Sertoli cells of the testis. In addition, the same study reported the destruction of the seminiferous tubules of mice after ZIKV infection (Govero et al., 2016).

## Zikv Determinants in Mosquitoes and Human

The evolution of virus–host interaction for survival is an arms race. While hosts have developed multiple mechanisms to protect themselves from infection, viruses generated diverse strategies to evade hosts’ defenses. Studies have shown that hosts can undergo genetic changes to develop defensive network through innate and adaptive immune responses to adapt and resist to viral infections (Barber, 2001; Martins et al., 2014). For instance, *Drosophila melanogaster* have shown to possess higher survival rate against Drosophila C virus infection after reaching at approximately 20th generation of progenies (Martins et al., 2014). Likewise, viruses possess the capability to undergo genetic changes to enhance viral replication in hosts (Agudelo-Romero et al., 2008; Tsetsarkin et al., 2014; Plauzolles et al., 2015).

The reason for the sudden emergence and wide spread of ZIKV remains elusive. In 2016, Weaver et al. (2016) hypothesized that an evolution in ZIKV adaptation to its mosquito vector has led to efficient transmission of the virus by *Aedes* mosquitoes. A similar situation had been observed in Chikungunya virus (CHIKV; Tsetsarkin et al., 2014). CHIKV, an arbovirus, was shown to undergo a series of mutations leading to the substitution of envelope glycoprotein. This in turn enhanced viral transmission as CHIKV could infect *Aedes albopictus* mosquitoes efficiently, leading to the dramatic spread of CHIKV in the Indian Ocean Basin, Asia, and Europe since 2005 (Tsetsarkin et al., 2014). Therefore, the sudden onset of the Zika outbreaks has raised questions on the genetic evolution of ZIKV.

To understand the evolutionary pattern of ZIKV, a whole genome comparative analysis of ZIKV was performed by comparing the pre-endemic (isolated prior to year 2007) and recent endemic ZIKV strains (Zhu et al., 2016). Interestingly, several changes were shared among the recent endemic ZIKV strains but not the pre-endemic ZIKV. According to Zhu et al. (2016) nine nucleotides that used to be located at the 3’ UTR stem loop II region of the pre-epidemic ZIKV strain was shown to be more closely resembling the stem loop I of epidemic strain. This structural change might be one of the reasons related to the increase transmissibility and virulence of the recent ZIKV (Zhu et al., 2016). Additionally, a total of 15 amino acid substitutions were detected in the endemic strains and most of these substitutions were located at the viral structural proteins (capsid, pre-membrane, and envelope proteins; Zhu et al., 2016). Molecular structure of envelope proteins, the largest proteins covering the virus surface area, was shown to be essential in viral attachment, fusion, replication, survival, and determining the host and cell tropism (Modis et al., 2004; Chávez et al., 2010; Wen et al., 2018). A number of mutations, particularly V603I and D679E, located in the domain III of the envelope proteins might be the key-leading factor to the viral virulence as these mutations were not found in pre-epidemic strains. Likewise, a V153M substitution located in the prM region was observed in all ZIKV epidemic strains but not the pre-epidemic strains, indicating the importance of the mutation (Modis et al., 2004; Chávez et al., 2010). In 2016, Jia et al. (2016) have shown that a single point mutation (T45G) in capsid gene had resulted in the reduced virulence of JEV. Thus, it is possible that the five amino acids’ changes in the capsid region of Asian ZIKV strains (ancestor for endemic ZIKV strains) had increased the virulence of ZIKV.

Non-structural proteins of *Flavivirus* may contribute to the recent ZIKV outbreaks. Liu et al. (2016) demonstrated the enhanced viral acquisition of mosquitoes due to the presence of NS1 protein of *Flavivirus*. This is followed by another study reporting a spontaneous mutation in ZIKV NS1 protein, which led to increased antigenemia in human, and led to recent ZIKV outbreaks (Liu et al., 2017). In addition, a new fragment of genetic recombination was found at the NS2B coding region of the Asian lineage of ZIKV which is similar to Spondweni virus (Zhu et al., 2016). Although the function of the new genetic recombinant remains elusive, these molecular changes could lead to the increased of virulence, replication efficiency, and host tropism of ZIKV.

On the other hand, Butt et al. (2016) reported that ZIKV had evolved its codon usage patterns according to its host and vector. These changes would ensure the successful transmission between multiple hosts and vectors. It is hypothesized that during the chain of transmissions, the high selection pressure induced by *A. albopictus* (compared to human and *Aedes aegypti*) might have led to the induction of new mutations in ZIKV genome, which could have triggered the new onset of neurological disorder in human (Butt et al., 2016). At the moment, the limited number of known ZIKV genome isolated from mosquitos is the limiting factor halting the understanding of ZIKV determinant in its vector and host. This constitutes an important knowledge gap which warrants further investigations.

Several reasons can be responsible for the sudden increase of ZIKV outbreaks. Besides the potential adaptive evolution undergone by ZIKV to enhance viral replication in mosquitoes, ZIKV may have adapted to human, resulting in higher viremia in human. While the recent outbreaks were closely related to the Asian lineage (Faye et al., 2014; Butt et al., 2016), phylogenetic relationship analyses had shown that ZIKV nucleotide sequences isolated from human samples shared a greater homology to the P6-740 strain (Malaysia/1966) which was the sole mosquito (*A. aegypti*) strain available in the Asian lineage (Wang et al., 2016). This finding suggested that P6-740 strain was the ancestor responsible for the emergence of recent epidemics. However, data revealed that 34 amino acid changes were shared among the recent outbreaks (FSM, H/PF/2013, and Brazilian strains), but surprisingly these changes were not found in ZIKV derived from mosquito (Wang et al., 2016). The possible reasons include the transmission of ZIKV through other routes of transmission such as sexual transmission, which may have contributed to the wide spread of the disease. Study also showed that the codon usage of NS1 gene of ZIKV has evolved toward the preferences of *Homo sapiens* instead of its *A. aegypti* host (Freire et al., 2018). The enhanced viral adaptation in human cells could serve as an important factor in leading to the recent sudden onset of ZIKV.

## Mosquito Determinants of Zika Epidemic Behavior

According to the American Mosquito Control Association (AMCA), there are about 2700 species of mosquito worldwide. However, only a few mosquito species are significant pests of humans, whereas many others are quite obscure, with findings suggested their unique habitats compared to viral disease vectors. *Aedes* mosquitoes lay eggs on moist surface, soil, or in containers that catch rain water, such as treeholes, flowerpots, and tires. The eggs of *Aedes* mosquitoes survive drying and hatch once exposed to water. The adults feed principally in day time, especially in the morning and evening.

The geographical distribution of ZIKV is closely related to the distribution of *Aedes* mosquitoes, the principal vector of ZIKV transmission (Wikan and Smith, 2016). After its first isolation from *A. africanus* in 1948 (Dick et al., 1952), ZIKV was also isolated from *Aedes apicoergenteus* in 1969 (McCrae and Kirya, 1982). Between 1971 and 1980, ZIKV antibody was detected in human serum in Nigeria (Fagbami, 1979; Adekolu-John and Fagbami, 1983) and Gabon (Jan et al., 1978). In 2007, *A. albopictus* was first recognized as the vector of ZIKV transmission after the invasion of *A. albopictus* to Gabon (Grard et al., 2014). As reviewed by Vorou (2016), the spread of ZIKV within and outside Africa is mainly driven by various species of *Aedes* mosquitoes. A study on genetic relationships among viral strains from Africa reported that the genome of ZIKV has exhibited many recombination events in various *Aedes* mosquito species, including *Aedes dalzieli*, *A. aegypti*, *Aedes furcifer*, and *A. africanus* (Faye et al., 2014). The same group of researchers also discovered that a minor post translational modification of ZIKV surface protein has contributed to its competency to the *A. dalzieli* vector (Faye et al., 2014). Since 1968, the distribution of ZIKV has been expanding to Europe (Tappe et al., 2014) and equatorial Asia, including India, Malaysia, Singapore, Thailand, Vietnam, Japan (Marchette et al., 1969; Olson et al., 1981; Kwong et al., 2013; Kutsuna et al., 2014), and Australia (Pyke et al., 2014; Leung et al., 2015). The transmission of ZIKV Asian lineage in these regions has been attributed to *A. aegypti* and *A. albopictus* (Roth et al., 2014; Calvez et al., 2016), another important vector during the most recent ZIKV outbreak in Brazil (Marcondes and Ximenes, 2016; Petersen et al., 2016). Although ZIKV transmission also occurs in other *Aedes* species, the first large outbreak in humans on Yap Island in 2007 may not be attributed to *Aedes henselli*, despite being the most prevalent mosquito species identified on Yap Island (Duffy et al., 2009). Similarly, ZIKV was not detected in *A. henselli* during the epidemic occurred in French Polynesia in 2013 (Musso, 2015). Ioos et al. (2014) also reported the possibility of *Aedes polynesiensis* as the mosquito vector for Zika outbreak in French Polynesia.

As an efficient epidemic vector of ZIKV, *A. aegypti* has close associations with human populations, especially in urban areas. In addition, the unique blood-feeding behavior of *A. aegypti* involves multiple human hosts in a single gonotrophic cycle and further enhances the vector competency of this mosquito species (Gubler, 1998). In the mid 1990s, yellow fever and dengue fever were effectively managed by controlling the populations of *A. aegypti*. However, in the past 30 years, the resurgence of yellow fever in Africa and of Dengue and Zika fever worldwide have highlighted the drop in efficiency in mosquito population control (Gubler, 2004; Bouri et al., 2012; Ebi and Nealon, 2016).

Transovarial transmission of *Flavivirus* was reported in 1979 for YFV (Aitken et al., 1979). A recent publication reported two *Aedes* strains with high level of midgut infections by ZIKV, with highly disseminated infection of ovaries, also provided transovarial transmission of ZIKV in mosquitoes (Thangamani et al., 2016; Ciota et al., 2017; Li et al., 2017). This provides new insights into biological mechanisms of mosquito vectors, as an intermediate host, in conferring optimum conditions for ZIKV dissemination and transmission.

Extrinsic incubation period (EIP) is determined by the interval between the acquisition of pathogen by a mosquito and the ability of the mosquito to transmit the pathogen to the next host. EIP in mosquito was found to shorten with viremic blood meals with a higher viral titer (Gould et al., 1962). However, the EIP for ZIKV in *A. aegypti* remains elusive despite the recent outbreaks and extensive research activities. In addition, the mechanisms of infection and dissemination of other *Flavivirus* members, such as YFV and DENV, have been well studied with a number of reports explaining their viral tropism in mosquito cells (Doi et al., 1967; Takahashi and Suzuki, 1979; Leake and Johnson, 1987; Linthicum et al., 1996; Salazar et al., 2007). The presence of *Flavivirus* in various parts of mosquitoes, including midguts, hindguts, legs, salivary glands, ovaries, compound eye, and central nervous system (Leake and Johnson, 1987; Mourya and Mishra, 2000), constitutes important research gaps which warrants further investigations for ZIKV transmission in mosquitoes.

## Vector Competence of Zika Virus Transmission

*Aedes* genus, most notably *A. aegypti* and *A. albopictus*, has been demonstrated to be the primary mosquito vectors for ZIKV (Weinbren and Williams, 1958; Wang et al., 2016; Gendernalik et al., 2017). However, little is known about the transmission of ZIKV via other mosquito genera. From the 1700 mosquito pools (a total of 11,247 mosquitoes) collected by Diallo et al. (2014) at the southeastern Senegal region, 31 samples were found positive for ZIKV. Interestingly, data showed that ZIKV was able to infect nine other *Aedes* species other than *A. aegypti*. This included the *A. furcifer*, *Aedes luteocephalus*, *A. africanus*, *Aedes vittatus*, *Aedes taylori*, *A. dalzieli*, *Aedes hirsutus*, *Aedes metallicus*, and *Aedes unilinaetus*. In addition, ZIKV was also found positive in *Mansonia uniformis*, *Culex perfuscus*, and *Anopheles coustani* mosquitoes. Furthermore, the vertical transmission of ZIKV by *A. furcifer* indicated the competency of this mosquito species as an important vector to maintain the circulation of ZIKV in mosquitoes (Diallo et al., 2014). Ledermann et al. (2014) have shown that ZIKV is able to infect *Aedes hensili*, indicating the potential role of the species in contributing to the viral transmission during outbreaks. In a recent review, Braack et al. (2018) have summarized a vast number of *Aedes* species (including *Aedes jamoti*, *Aedes opok*, *Aedes flavicollis*, *Aedes graham*, *Aedes taeniorostris*, *Aedes tarsalis*, *A. vittatus*, *Aedes dalziella*, *Aedes fowleri*, *Aedes minimus*, and *Aedes neoafricanus*) and some less common vectors including *Anopheles gambiae*, *Eretmapodites inornatus*, and *Eretmapodites quinquevittatus*, to be ZIKV competent. On the other hand, *A. gambiae*, *Anopheles stephensi*, and *Culex pipiens* mosquitoes were shown to be refractory to ZIKV infection (Dodson and Rasgon, 2017; Kenney et al., 2017). Dodson et al. (2018) also demonstrated that the ZIKV strain isolated from Puerto Rico outbreak in 2015 was unable to infect *Anopheles freeborni*, *Anopheles quadrimaculatus*, and *Culex tarsalis* mosquitoes which are predominantly circulate in North America. Interestingly, the wild-caught female *C. tarsalis* in Mexico has shown otherwise (Elizondo-Quiroga et al., 2018).

Controversial results were obtained in *Culex quinquefasciatus* species as a competent vector. Guo et al. (2016) have demonstrated the ability of ZIKV to infect the *C. quinquefasciatus* captured in urban areas of China. The study was supported by a later study where Guedes et al. (2017) reported the ability of ZIKV to infect the laboratory-reared *C. quinquefasciatus* and viruses were successfully isolated from the field-caught *C. quinquefasciatus*. A recent study by Elizondo-Quiroga et al. (2018) has also successfully isolated ZIKV from *C. quinquefasciatus* along with other species including *Aedes vexans*, *Culex coronator*, and *C. tarsalis*. However, surprisingly, many studies failed to demonstrate the competency of *C. quinquefasciatus* as a ZIKV transmission vector (Dodson and Rasgon, 2017; Duchemin et al., 2017; Kenney et al., 2017; Roundy et al., 2017), including a recent review by van den Hurk et al. (2017) which stated that most populations of *C. quinquefasciatus* were refractory to ZIKV infection. When challenged with ZIKV strain of Cambodia 2010 origin, *Culex annulirostris* and *C. quinquefasciatus* mosquitoes were shown to be refractory to ZIKV infection whereby no ZIKV was detected in saliva, midgut, and carcass via qRT-PCR and TCID_50_ (Duchemin et al., 2017). Meanwhile, Lourenço-de-Oliveira and Failloux (2017) had summarized the competency studies done on the eight *Culex pipens* and 10 *C. quinquefasciatus* populations across five continents, and both species were shown to be incompetent in transmitting ZIKV in all studies. Therefore, careful interpretations and further studies are required to examine the competency of *C. quinquefasciatus* as a transmission vector as many experimental studies have suggested otherwise. Systematic review study has indicated that *A. aegypti* and *A. albopictus* were the predominant vectors for ZIKV, while *Culex*, *Anopheles*, and most *Aedes* species were generally observed to be refractory to ZIKV infection (Epelboin et al., 2017).

## Mosquito Immune System Against Zika Virus Infection

Despite the major concern of global health and significant economic losses, some of the mosquito-borne viral diseases are still being neglected. Mosquitoes are very permissive to some important arboviruses which render them an important vector in transmitting these viruses. However, these viruses neither result in dramatic pathological conditions nor impair the lifespan of mosquitoes. Once a mosquito is infected with an arbovirus, it remains infectious throughout the whole lifespan. As the transmitting vector, mosquitoes provide optimal conditions that allow rapid replication of arboviruses, from the midgut to the hemolymph, subsequently into the fat body, muscles, neural tissue, and salivary glands (Girard et al., 2004; Romoser et al., 2004; Salazar et al., 2007; McElroy et al., 2008).

Among several conserved innate immune responses in systemic antiviral strategies in mosquitoes, RNA interference (RNAi) mechanism remains the most heavily relied intracellular antiviral mechanisms, which have been comprehensively studied to limit viral propagation in insect vectors (Keene et al., 2004; Wang et al., 2006; Sánchez-Vargas et al., 2009; Khoo et al., 2010; Arjona et al., 2011; Cheng et al., 2016). This section focuses on the possibility of ZIKV regulation in mosquitoes using RNAi system.

Despite the same viral family – *Flaviviridae*, DENV and ZIKV may not share similar infection routes in *Aedes* mosquitoes (Oliveira et al., 2017). In addition, the persistent mutations discovered in ZIKV were reported to inhibit cellular antiviral activities by altering the secondary structure of ZIKV RNA genome (Yokoyama and Starmer, 2017). However, although limited study on the role of mosquito RNAi mechanism is available, scientists believe that RNAi and micro-RNA play crucial roles in ZIKV regulation (Saldaña et al., 2017). This is also supported by a recent publication of mosquito symbiont-mediated RNAi delivery system using *Rhodnius prolixus* and *Frankliniella occidentalis*. These bacteria can be manipulated to deliver dsRNA and, when ingested, able to compete with wild-type microflora in mosquito midgut, while mediating systemic knockdown phenotypes that were transmissible (Whitten et al., 2016). These RNAi delivery systems using *R. prolixus* and *F. occidentalis* could be adapted in mosquito vectors of ZIKV to further investigate the roles of miRNA in managing the replication of ZIKV in *Aedes* mosquitoes.

In addition to RNAi, the evolutionarily conserved pathways such as Toll and Imd pathways are also crucial in regulating arbovirus infection in insects, especially in *Drosophila* (Tsai et al., 2008; Costa et al., 2009). It is notably that genomic analyses revealed some highly conserved regions of Toll and Imd genes between *Drosophila* and mosquitoes (Waterhouse et al., 2007; Bartholomay et al., 2010). In addition, some reported the antiviral properties of *Aedes* Toll or Imd pathways on other arboviruses (Xi et al., 2008; Sim and Dimopoulos, 2010; Luplertlop et al., 2011; Carissimo et al., 2015). However, the antiviral function of these mosquito immune mechanisms against ZIKV remains elusive.

The 5’ and 3’ untranslated regions of ZIKV were reported to play essential roles in viral RNA replication, viral transmissibility, and host immune modulations (Ng et al., 2017). A recent study described the possible role of ZIKV non-coding RNAs in confounding a cellular exonuclease (Akiyama et al., 2016), which was in line with some previous studies reporting the role of *Flavivirus* UTRs in suppressing RNAi machinery in the vectors (Eulalio et al., 2007; Pijlman et al., 2008; Funk et al., 2010). These findings were supported by another group of researchers describing the role of non-coding *Flavivirus* RNA in displaying RNAi suppressor activity in their vector and host cells (Schnettler et al., 2012). In addition, a later publication described the role of YFV capsid protein in suppressing mosquito RNAi mechanism (Samuel et al., 2016). These evidences support the hypothesis that ZIKV, as a newly emerged *Flavivirus*, can circumvent the RNAi mechanism in mosquito cells, although some hypothesized that arboviruses may not need an RNAi suppressing system in order to establish a persistent infection of the insect host (Umbach and Cullen, 2009).

To date, there is no report on the elucidation of mosquito defense mechanism against ZIKV infection. The knowledge gaps between mosquito innate immune response to ZIKV infection remained elusive.

## Research Gaps

A strong research attention on ZIKV can be reflected by a recent PubMed search, with a total of 216 articles published between 1952 and 2015, to an annual publication of 1718 and 1881 articles in the year 2016 and 2017, respectively. Majority of these articles focus on surveillance studies, which limited to certain regions where data may not be applicable to others. In addition, many researchers focus on the mode of transmission between mosquitoes and vertebrates, including humans, and the natural history, diagnostics, epidemiology, or clinical manifestations of ZIKV infections. This section suggests several research areas that need more focus in the field of ZIKV research.

ZIKV causes severe neurologic complications – Guillain-Barré syndrome and microcephaly in unborn babies (Oehler et al., 2014; Gonzalez-Escobar et al., 2018). These important clinical features gained awareness in the Geneva meeting to highlight the urgent needs in obtaining a better understanding of the associated illness and clinical manifestations, strategies in vaccine/drug design, and development of effective diagnostic tools and vector control. In addition, since the ZIKV outbreak in 2015, several WHO meetings have emphasized the lacking of evidence on the effectiveness of the current vector-control intervention strategies, such as mass spraying of insecticides, in controlling the spread of arbovirus transmission. Although the anti-ZIKV effect of suramin was recently reported (Albulescu et al., 2017), the existing knowledge on viral tropisms in mosquitoes and the role of various mosquito organs in the transmission of ZIKV remained elusive.

Mosquito vectors ingest infectious viral particles into the midgut during a viremic blood meal. Following infection of midgut cells, mature arboviral particles are disseminated from the midgut and ultimately to the salivary glands for an effective infection, followed by salivary secretion to the subsequent hosts. These processes were well studied in other members of *Flaviviruses* or mosquito-borne viruses (Zhang et al., 2010; Tham et al., 2014, 2015; Cime-Castillo et al., 2015; Kantor et al., 2017; Valderrama et al., 2017), but relatively less efforts are found on ZIKV. In addition, multiple infection of arboviruses (Rückert et al., 2017) and transovarial dissemination of ZIKV should gain more attentions, which can be exploited for novel biologic and genetic control strategies.

Manipulation of mosquito cellular machineries, such as influencing the normal RNAi systems, has been shown effective in reducing vector compatibility to arboviruses (Qsim et al., 2017; Terradas et al., 2017). Several recent techniques such as sterile insect technique (Franz et al., 2014; Qsim et al., 2017) or obligatory intracellular *Wolbachia* (Ye et al., 2015; Terradas et al., 2017) have also been shown successful in reducing the vector competence of *A. aegypti*. However, concerns were raised on the effectiveness, stability, and loss of virus resistance phenotype in mosquito vectors over time (Franz et al., 2009; Wilke et al., 2018), and the efficiency of these novel approaches in managing the transmission of ZIKV requires more investigations.

In contrast to severe disease manifestations observed in vertebrates, mosquitoes evolved to control viral tropisms and replication to a non-pathogenic level without compromising their fitness throughout the lifespan, while allowing efficient viral transmission from one host to another (Hegde et al., 2015; Cheng et al., 2016). A recent publication on transcription profiling of defensins of *A. aegypti* has suggested differences in antiviral defense response when mosquito was exposed to CHIKV and ZIKV (Zhao et al., 2018) Therefore, subsequent studies should also specifically focus on vector immunity-ZIKV interplay to deepen the understanding of ZIKV tropism, dissemination, and replication in mosquito vectors.

## Conclusion

Among all the economically important arboviruses, research focus on ZIKV tropism and transmission in mosquito cells is still lacking. As supported by the recent “Zika Virus Research Agenda” by WHO, continuous research attentions with sustainable resources are needed to support the discovery of novel molecular interactions, such as protein–protein interactions or protein–nucleic acid interactions, between ZIKV and *Aedes* mosquitoes. In addition, rapid and efficient feedbacks of such research activities are needed to nurture and support the development of novel strategies/tools in vector control.

## Author Contributions

The collaborations between all four authors helped in developing the idea about the review paper. H-WT structured the manuscript, did the majority of the writing, and continuously received comments and amendments from VB, MO, and M-FC throughout the writing process, including several meetings with all four authors present. The manuscript has been finalized and checked by all four authors prior to submitting.

## Conflict of Interest Statement

The authors declare that the research was conducted in the absence of any commercial or financial relationships that could be construed as a potential conflict of interest.
